# Lynch Syndrome from a surgeon perspective: retrospective study of clinical impact of mismatch repair protein expression analysis in colorectal cancer patients less than 50 years old

**DOI:** 10.1186/1471-2482-14-9

**Published:** 2014-02-17

**Authors:** Gian Luca Baiocchi, Nazario Portolani, William Vermi, Carla Baronchelli, Federico Gheza, Claudio Zogno, Alessandro Scaglia, Eleonora Marchina, Guido AM Tiberio, Stefano Maria Giulini

**Affiliations:** 1 Department of Clinical and Experimental Sciences, Brescia University, Brescia, Italy; 2 Department of Pathology, Brescia University, Brescia, Italy; 3 Genetic and Biology, Department of Biomedical Sciences and Biotechnologies, Brescia University, Brescia, Italy

## Abstract

**Background:**

In clinical practice, unexpected diagnosis of colorectal cancer in young patients requires prompt surgery, thus genetic testing for Lynch Syndrome is frequently missed, and clinical management may result incorrect.

**Methods:**

Patients younger than 50 years old undergoing colorectal resection for cancer in the period 1994-2007 were identified (Group A, 49 cases), and compared to a group of randomly selected patients more than 50 (Group B, 85 cases). In 31 group A patients, immunohistochemical expression analysis of MLH1, MSH2 and MSH6 was performed; personal and familial history of patients with defective MMR proteins expression was further investigated, searching for synchronous and metachronous tumors in probands and their families.

**Results:**

Fifty-one percent of patients did not express one or more MMR proteins (MMR-) and should be considered Lynch Syndrome carriers (16 patients, group A1); while only 31.2% of them were positive for Amsterdam criteria, 50% had almost another tumor, 37.5% had another colorectal tumor and 68% had relatives with colorectal tumor. This group of patients, compared with A2 group (< 50 years old, MMR+) and B group, showed typical characteristics of HNPCC, such as proximal location, mucinous histotype, poor differentiation, high stage and shorter survival.

**Conclusions:**

The present study confirms that preoperative knowledge of MMR proteins expression in colorectal cancer patients would allow correct staging, more extended colonic resection, specific follow-up and familial screening.

## Background

Hereditary non-polyposis colorectal cancer (HNPCC), also known as Lynch Syndrome [1], is an autosomal-dominant syndrome accounting for 3-5% of colorectal cancer cases [2,3], predisposing to female reproductive and urinary tract cancers, as well as other extracolonic tumors [4,5]. It is actually known that Lynch Syndrome is caused by an inherited or acquired mutation that inactivates one or more genes for the mismatch repair (MMR) proteins, which normally recognize the wrongly incorporated nucleotide and replace it with the correct one, leading to accumulation of cellular mutations and thus greatly increasing the likelihood of malignant transformation [6,7]. Almost five DNA MMR genes (MLH1, MSH2, MSH6, PMS2 and PMS1) have been individuated as possible mutation sites, and their proteins unexpression or mutation, have been investigated as possible causes of Lynch Syndrome. Patients with MMR genes mutation and protein absence or inactivation have a lifetime risk of CRC of 70-80% if no regular endoscopic screening is performed, with a mean age of CRC diagnosis of 44 years [4].

The diagnosis of Lynch Syndrome generally requires three steps, including a review of family history of cancer, the cancer specimen analysis and the genetic analysis, i.e. the determination of the primary DNA sequence of MMR genes [8-10]. In clinical practice, however, outside the families known to be affected by Lynch Syndrome, the most frequent situation is the unexpected diagnosis of colorectal neoplasm in a young patient, for which a treatment program should be scheduled as soon as possible, not allowing a proper genetic study before surgery. In these cases, if the patient is not correctly diagnosed as a Lynch Syndrome bearer, treatment may finally result inappropriate in terms of completeness of preoperative staging and extension of colonic removal.

The aim of the present study is to determine if a preoperative diagnosis of HNPCC by MMR proteins expression analysis could affect treatment decisions, leading to a better management.

## Methods

All colorectal resections for cancer performed at the Surgical Clinic of the University of Brescia in the period 1994-2007 were retrospectively analyzed. Every patient entered into a scheduled program of clinical and instrumental follow-up. Our object was the comparison between patients under 50 years (Group A), and a sample of 85 randomly selected patients (which follow-up was recently updated for another study), more than 50 (included) years old (group B). The following parameters were recorded: age, sex, clinical presentation (symptomatic or not), operative treatment (resection of a colonic segment or extended surgery, including at least two segments or subtotal/total colectomy), cancer site (proximal to the transverse colon included, or distal), histotype (mucinous or not), staging (T1-2 or T3-4), grading (G1-2 or G3), disease-free and overall survival rates.

In group A, immunohistochemical analysis on histological samples derived from preoperative endoscopic biopsies and/or removed pieces was performed, to determine the loss of expression of 3 MMR proteins (MLH1, MSH2 and MSH6). After retrieving the paraffin blocks, 8 micron thick sections were obtained and stained with hematoxylin and eosin. The slides were placed in a microwave oven for 5 minutes in jet mode (full power) twice, and 3 more times for 5 minutes at 750 W power and lastly incubated for 40 minutes. Anti-MLH1 Mouse antibody clone G168-15 BD, at 1:30 dilution (Biosciences CompanyTM, Toronto, Ontario, Canada, code 551092), anti-MSH2 Mouse antibody, clone G216-1129, at 1:20 dilution (Biosciences CompanyTM, Toronto, Ontario, Canada, code 556349) and the Anti-MSH6 Mouse antibody, clone 44 (Zymed Laboratories Inc.TM, San Francisco, California, USA, code 08-1374) have been employed. For antigen unmasking the EDTA buffer at pH 8, 0.1 M was used. Data about MMR were expressed as dichotomous variables (yes/no), even though in 8 cases a diagnostic doubt was due to an insufficient but not entirely absent expression of the protein. The cases with preserved expression were considered normal, those without expression (as well as those with diagnostic doubt) were not. In the present work it was not taken into account what has been previously demonstrated: the absent expression of MSH6 is associated in 5% of cases with impaired quality (but with normal immunohistochemical expression) of MSH2, in which case it would be useful to proceed with its genetic analysis [11-13]. Furthermore, in the present work we did not investigate micro satellite instability (MSI) and gene sequences of MMR.

Patients with complete or partial lack of expression (group A1) were compared to patients with preserved expression (group A2) of one or more MMR protein, concerning clinical, pathological and survival features. In patients belonging to group A1, the personal and family history was deeply assessed, up to the previous second generation (brethren, parents, uncles, grandparents and grandparent’s brethren) and first subsequent generation (children and brethren’s children), in accordance with the Amsterdam II criteria [14]; for patients in which such informations were not clearly available from the analysis of medical supplies, telephone interviews were conducted. All the relatives in the 4 generations and the probands were investigated for every type of cancer, including previous and metachronous ones; colorectal polyps were considered as neoplasm if high-grade dysplasia was documented. All the interviews were done in 2010, from March to July, follow-up information being recorded up to 12/12/2009.

Statistical analysis was performed by Chi square test for dichotomous variables and Student's t test for continuous variables. Survival curves were constructed with the actuarial model, and comparison between curves was carried out by the method of Mantel-Haenzn.

This study was approved by the University Institutional Board Ethics Committee (Department of Clinical and Experimental Sciences).

## Results

**Figure 1:**
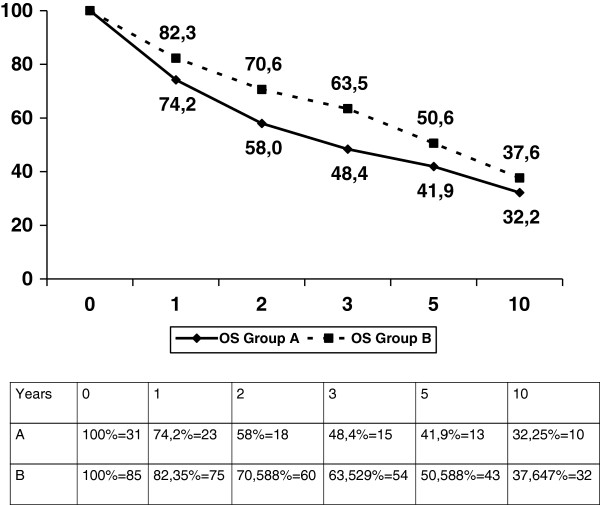
A vs B overall survival (p = NS).

The entire series of colorectal resections for cancer in the period under investigation consists of 814 patients. Forty-nine patients less than 50 years old were identified (group A), with a mean age of 41 years; 27 were males and 22 females. Table 1 compares clinical and pathological features of Group A patients with those of patients older than 50 years (Group B); the former had a more frequently symptomatic tumor, the most reported symptoms being abdominal pain (43% of cases), bleeding (35% of cases), occlusion (14%) and palpable abdominal mass (8% of cases). Moreover, more undifferentiated tumors and advanced stages were identified in group A patients; however, the prognosis was not significantly different (Figure 1).

**Table 1:** **Comparison between group A (<50 years) and group B (>** **50 years).**

	**Group A (49)**	**Group B (85)**	**p**
Age (mean)	40,2	66,0	0.001
Symptoms	93,8%	62,3%	0.031
Extended surgery*	9,7%	9,4%	0.965
Proximal localization**	38,7%	34,1%	0.647
Mucinous	16,1%	14,1%	0.786
Advanced stage (T3-4)	61,3%	37,6%	0.023
Undifferentiated	32,2%	9,4%	0.0026
Disease free survival (mean)	73 m	81 m	0.78
Overall survival (mean)	79 m	89 m	0.86

*More than 1 segment colonic resection.

**Proximal to the transverse colon, included.

In group A, 31 wax blocks were retrieved from 49 patients; in 3 cases a final diagnosis of familial adenomatous polyposis (FAP) was confirmed by genetic testing, while the remaining 15 cases were not available for reevaluation or were used for other investigations. In 8 patients out of 31 available cases, both endoscopic biopsies and surgical specimens were analyzed, and the 2 determinations were always concordant. Fifteen patients had a normal expression of MLH1, MSH2 and MLH6, 8 patients had a doubtful expression (25.8%), and 8 patients showed markedly deficient expression of at least one MMR protein (25.8%). The clinical features of the 16 patients considered as having altered immunohistochemical expression of MMR protein(s) are as follows: 5/16 patients (31.2%) belonged to Amsterdam and 16/16 patients belonged to Bethesda criteria (due to the inclusion in group A of only younger than 50 year old cases) [15,16]; 8/16 patients (50%) had an association with other synchronous or metachronous extracolonic tumors; in 11/16 cases (68.7%) another colorectal cancer developed in the 4 generations analyzed for each patient; finally, 6/16 patients (37.5%) developed another subsequent colonic neoplasm (Table 2). A total of 37 cases of cancer were diagnosed, including colorectal (19), endometrium (6), breast (2), kidney (2), Vateri papilla (1), prostate (3), skin cancers (3) and lung (1).

**Table 2:** Deficiency of mismatch repair protein expression (group A1)

**Case**	**MLH1**	**MSH2**	**MSH6**	**AMST**	**BETH**	Other tumors*	Familial history**	**Colon recurrence**
1	-	+	-	YES	YES	NO	YES	YES
2	-	+	+	NO	YES	YES	YES	YES
3	+	-	+	NO	YES	NO	NO	NO
4	+	+	-	NO	YES	YES	YES	NO
5	+	+	-	NO	YES	YES	NO	NO
6	-	+	+/-	NO	YES	NO	YES	YES
7	+	-	+	YES	YES	NO	NO	NO
8	-	+/-	+	NO	YES	NO	YES	NO
9	+	+	+/-	NO	YES	YES	YES	NO
10	+/-	+	+/-	YES	YES	NO	NO	NO
11	+	+	+/-	NO	YES	YES	YES	YES
12	+/-	+	+/-	YES	YES	NO	NO	NO
13	+/-	+	+	NO	YES	YES	YES	YES
14	+	+	+/-	NO	YES	YES	YES	YES
15	+	+	+/-	NO	YES	YES	YES	NO
16	+	+	+/-	YES	YES	NO	YES	NO

+/- Partial expression of the protein.

*Both synchronous and metachronous.

**Four generations have been analyzed (see the text).

**Figure 2:**
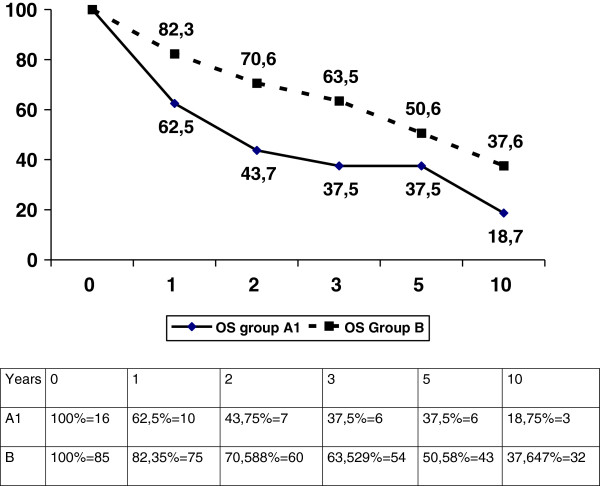
A1 versus B overall survival (p = 0.044).

Table 3 reports the comparison between HNPCC cases (group A1) and general population (Group B). In group A1 the following clinical presentations were found: high percentage of proximal, mucinous, poorly differentiated and high stage tumors. The survival was significantly lower for Group A1 than for Group B (p = 0.048, Figure 2) and group A2 (p = 0.049) patients.

**Table 3:** Comparison between group A1 (<50 years, MMR-, thus HNPCC cases) and group B (> 50 years)

	**Group A1 (16)**	**Group B (85)**	**Group A2 (33)**	**P**
Age (mean)	40	66		0.001
Symptoms	100%	62%		0.031
Extended surgery*	43.7%***	9,4%		0.032
Proximal localization**	43,7%	34,1%		0.46
Mucinous	56.2%	14,1%		0.047
Advanced stage (T3-4)	75%	36,4%		0.0042
Undifferentiated	31,2%	9,4%		0.0167
Disease free survival (mean)	63,9 m	81 m		0.057
Overall survival (mean)	64,5 m	89 m		0.048
Disease free survival (mean)	63,9 m		78,2 m	0.061
Overall survival (mean)	64,5 m		81,6 m	0.049

*More than 1 segment colonic resection.

**Proximal to the transverse colon, included.

***Including resections for metachronous tumors.

## Discussion

Colorectal cancer in Lynch Syndrome have distinctive clinical features that are well known in literature, such as younger age at presentation, proximal location, mucinous differentiation, advanced stage, associated extracolonic tumors and better stage-normalized prognosis [2,6,17]. However, even if well-known management guidelines are available, in daily clinical practice most frequently a diagnosis of colon cancer in a young patient is not immediately followed by a routine assessment for Lynch Syndrome - except in cases in which the diagnosis is made during a screening program in a family known for bearing the genetic mutation - thus patient does not receive specific treatment. In particular, the surgeon may not perform a more extensive surgery on the colon (total or subtotal colectomy [18]), eventually associated in women with prophylactic hysterectomy or hystero-ovariectomy, and may not look for synchronous extracolonic tumors [19,20]. At most, the study is left to postoperative time. The main reason of this common practice is related to the delay of cure required to carry out proper genetic investigations. In fact, as demonstrated by this series of patients undergoing colonic resection for cancer before 50 years of age, in young patients the cancer is often symptomatic for abdominal pain, bleeding, obstruction or palpable abdominal mass (over 90% of our patients). Furthermore, waiting few weeks may be not tolerable from a psychological point of view. Thus, our series represents the current clinical practice, in which most frequently a correct recognition of Lynch Syndrome failed.

Compared to patients older than 50 years, young patients (Group A) showed a statistically significant difference in the rate of undifferentiated and high stage tumors (Table 1). This is easily explained by the fact that colon cancer diagnosis are often late in young people, in a symptomatic stage. Accordingly, patients in group A seem to have a worse prognosis (not statistically significant, Figure 1). Other typical features of Lynch Syndrome (mucinous histology and proximal location), while more frequent, were not significantly represented in this group. The proportion of patients undergoing extensive surgery was also similar in groups A and B.

The aim of the present work was therefore to analyze the expression of MMR proteins in young patients by immunohistochemical determination of MLH1, MSH2 and MSH6, in order to identify those actually belonging to a Lynch Syndrome family.

A limited sample of 31 cases was available for the analysis. Out of these, 16 had a clearly deficient or absent expression of at least one MMR protein, and thus were HNPCC-related colorectal cancer cases (Group A1). This group is indeed more homogeneous than the whole group A regarding clinical characteristics of malignancies, as shown in Table 3: these patients had more frequently a proximal and mucinous tumor, and their prognosis was significantly worse than for group B (Figure 2); this is partly in disagreement with the literature, and may be explained by two reasons: first, all group A1 patients were diagnosed because of symptoms, thus with an advanced stage, while the data reported in the literature usually refer to patients known to be affected by Lynch Syndrome, thus including a relevant proportion of cases diagnosed through screening investigations; second, and perhaps more important, none of these patients received proper treatment, including early research and treatment of other cancers and extended colectomy to prevent colon cancer recurrence. Indeed, the difference in survival between A1 (< 50 years, MMR-) and A2 (<50 years, MMR+) remains significant because those groups are more homogenous, being both very young and having both a great proportion of advanced symptomatic cases.

Analyzing in detail the clinical history, before and after the intervention, half of these 16 patients did previously have or subsequently developed other tumors; in particular 37.5% developed metachronous colonic tumors requiring redo-surgery. Only 1 out of 16 MMR deficient patients underwent a primary extended colectomy; 5 our of 15 patients receiving primary segmental resection subsequently developed a colonic recurrence. Prophylactic subtotal colectomy may be proposed as alternative to surveillance colonoscopy for individuals with confirmed mutations. Syngal and Collegues used a decision-analysis model to evaluate life expectancy and quality-adjusted life expectancy in 25 years old patients with a confirmed mutation undergoing prophylactic surgery, compared with colonoscopy surveillance. They showed that, although both approaches offer only a modest survival benefit, immediate procto-colectomy or subtotal colectomy was superior to surveillance, with an expected gain in life expectancy of 15.6 and 15.3 years after the immediate proctocolectomy or subtotal colectomy respectively, compared with 13.5 years for surveillance [21]. In a recent paper, Parry and Colleagues found that 0% and 22% respectively of patients receiving extended and segmental colonic resection had a metachronous colorectal cancer, with a risk reduction of 31% (95% CI 12% to 46%; p = 0.002) for every 10 cm of bowel removed [18].

These data clearly show at what extent a correct recognition of Lynch Syndrome may influence the management of affected patients, both in terms of diagnostic approach (including preoperative staging and follow-up) and surgical strategy. On the other hand, clinical diagnosis based on medical history, including a precise picture of the genealogical tree for at least four generations, referring to the Amsterdam and/or Bethesda criteria, seems from our study to be inadequate: we identified only 31% of HNPCC patients by Amsterdam criteria, while only about half of those who fulfilled Bethesda criteria (i.e. all our patients Group A) were affected by HNPCC. The immunohistochemical determination of MLH1, MSH2 and MSH6 proteins has been simple, cheap and fast in this series. Moreover, according to our data, the biopsy samples can be used for this purpose in the preoperative period. In all the 8 cases in which the analysis was done both on preoperative biopsies and surgical specimen, there was a complete agreement in MMR proteins expression. Similar results have been recently reported by Warrier and Colleagues in a series of 33 germ-line positive mutation carriers patients in which the sensitivity of the endoscopic biopsy in predicting the germ-line status was 94.9% (95% CI 80.4-98.3) [22].

Lack of PMS2 immunohistochemical analysis and MSI determination are main limitations of the present study; however, some recent proposal to reduce the number of MMR proteins analysis to lower costs in daily practice is appearing; for instance, Shia and Collegues provided evidence that a 2-antibody panel is as effective as the current 4-antibody panel in detecting DNA mismatch repair protein abnormalities [23]. MSI is a very useful diagnostic tool, complementary to the immunohistochemical determination of MMR proteins expression, and it can be conducted rapidly on the biopsy material too. Furthermore, MSI analysis has a 93% sensitivity in detecting MMR deficiency in MMR mutation carriers [24]. However, compared with MSI analysis, immunohistochemistry has the additional advantage of identifying the MMR gene which is most eligible for DNA analysis [25]. On the other hand, MMR proteins belong expressed in case of protein truncation, such as frame shift, splice site mutations and large genomic rearrangements: the abnormal protein may have a loss of function but be still detected with immunohistochemisty [11-13]. Furthermore, this work did not provide an implementation of genetic testing, which is considered time-consuming in the preoperative setting [15].

## Conclusions

The retrospective analysis of a 20 years series of colonic resections for malignancy, specifically focused on patients which were diagnosed to have a colorectal cancer less than 50 years old, confirms that the preoperative identification of patients with Lynch Syndrome, by the immunohistochemical determination of the MMR proteins expression, may have a great immediate clinical relevance [26], leading to a correct surgical and global care strategy.

## Competing interest

The authors declare that they have no competing interests.

## Authors’ contributions

Study concept and design, GLB and CB; acquisition of data, FG, CZ, EM and AS; analysis and interpretation of data, WV; drafting of the manuscript, GLB and NP; critical revision of the manuscript for important intellectual content, GT and SMG. All authors read and approved the final manuscript.

## Pre-publication history

The pre-publication history for this paper can be accessed here:

http://www.biomedcentral.com/1471-2482/14/9/prepub
